# The prognostic impact of preoperative nutritional status on postoperative complications and overall survival in patients with resectable pancreatic cancer

**DOI:** 10.1007/s00520-025-09303-1

**Published:** 2025-03-03

**Authors:** Elif Gündoğdu, Betül Nalan Karahan, Ahmet Murat Şendil, Akile Zengin, Murat Ulaş, Mehmet Kılıç

**Affiliations:** 1 Department of Radiology, Faculty of Medicine, Eskişehir Osmangazi University, Meşelik Yerleşkesi, 26480 Eskişehir, Turkey; 2 Department of General Surgery, Faculty of Medicine, Eskişehir Osmangazi University, Eskişehir, Turkey

**Keywords:** PNI, HALP score, Sarcopenia, Osteoporosis, Pancreatic cancer

## Abstract

**Purpose:**

The aim of the study is to demonstrate the prognostic impact of preoperative nutritional status indicators, including prognostic nutritional index (PNI), hemoglobin-albumin-lymphocyte-platelet (HALP) score, sarcopenia index (SI), and bone mineral density (BMD) values, on postoperative complications and survival in patients with resectable pancreatic cancer (PC).

**Materials and methods:**

The medical data and computed tomography (CT) images of 85 patients who undergone surgery for PC between January 2017 and 2023 were evaluated retrospectively. Patients were grouped according to the presence or absence of sarcopenia and osteoporosis, high and low PNI and HALP scores. The groups were compared in terms of the complication development rate, 30- and 90-day mortality, and 5-years overall survival (OS). Sarcopenia and osteoporosis were determined from CT images (sarcopenia index used for sarcopenia, bone mineral density for osteoporosis).

**Results:**

Except from OS (*p* < 0.0001), no differences were found between sarcopenic and non-sarcopenic groups in terms of postoperative complications, 30- and 90-day mortality (*p* = 0.775, *p* = 0.704, *p* = 0.196, respectively). There were no differences between the groups with and without osteoporosis in terms of the presence of postoperative complications, 30- and 90-day mortality, and OS (*p* = 0.770, *p* = 0.608, *p* = 0.196, *p* = 0.09, respectively) as low and high HALP score groups (*p* = 0.236, *p* = 0.696, *p* = 0.299, *p* = 0.45, respectively). Except from a 30-day mortality (*p* = 0.03), no differences were found between low and high PNI groups in terms of postoperative complications, 90-day mortality, and OS (*p* = 0.82, *p* = 0.09, *p* = 0.18, respectively).

**Conclusion:**

PNI may be used as prognostic data for early postoperative mortality, while sarcopenia may be indicative of 5-year OS in patients with resectable PC. Our results suggest that providing nutritional support may potentially improve prognosis. Future studies, in which other factors effective in prognosis are evaluated together with nutritional status, will show more information on this subject.

## Introduction

According to the Global Cancer Statistics 2022, pancreatic cancer (PC) was the sixth leading cause of cancer-related deaths in both sexes and responsible for almost 5% of all cancer deaths worldwide [1]. Recently, while mortalities related to cancers such as lung, colorectal, prostate, breast, and stomach have decreased, mortality rates associated with PC have remained quite stable [1]. Despite the development of strategies to detect and manage PC, only 4% of patients remain alive for 5 years after diagnosis [2]. The primary reason for the poor prognosis of this disease is its late diagnosis. Surgical resection is still the only treatment providing prolonged survival [3]. However, approximately 85% of tumors are unresectable for cure at the initial staging assessment due to liver metastases, peritoneal seeding, or loco-regional extension [3]. Prognosis mainly depends on the resectability of the tumor, but it is not the only factor; prognosis can also vary in patients who undergo surgery. These factors can be related to the patient, the treatment, and tumor-specific reasons. One of the most important patient-related factors is nutritional status [4].

Various scoring systems have been developed to determine nutritional status. For this purpose, the prognostic nutritional index (PNI) was proposed as a marker by Buzby et al. in 1980 [5], but due to its subjective data, the formula was modified by Onodera et al. in 1984 [6], and a meta-analysis conducted in 2014 showed that a low PNI is a risk factor for poor prognosis in cancer patients [7]. The hemoglobin-albumin-lymphocyte-platelet (HALP) score developed by Chen et al. [8] has gained attention as a new prognostic biomarker for predicting immuno-nutritional status [9]. These scoring systems are simple and cost-effective laboratory parameters. Other markers that can determine nutritional status are sarcopenia and osteoporosis. Sarcopenia is a syndrome characterized by a decrease in skeletal muscle mass, which can be seen especially in cancer patients due to cachexia. Osteoporosis is characterized by a decrease in bone mass and increased bone fragility. Both sarcopenia and osteoporosis can be quantitatively determined by computed tomography (CT). CT is routinely used for preoperative staging in patients with PC. It is possible to determine the bone mineral density (BMD) values and sarcopenia index (SI) from this CT.

In this study, the aim is to demonstrate the prognostic impact of preoperative nutritional status indicators, including prognostic nutritional index (PNI), hemoglobin-albumin-lymphocyte-platelet (HALP) score, sarcopenia index (SI), and bone mineral density (BMD) values, on postoperative complications and survival in patients with resectable pancreatic cancer (PC).

## Materials and methods

The study was conducted in a tertiary-care hospital and approved by the Ethics Committee of the Faculty of Medicine of Eskişehir Osmangazi University (No: 58 Date:20.06.2023). The study was conducted in accordance with the principles of the Helsinki Declaration. Approval and informed consent were not necessary due to retrospective nature of study and were waived by our local institutional review board.

### Study population

The medical data of patients who underwent CT between January 2017 and January 2023 were evaluated retrospectively. Patients who had pancreatic mass on CT and undergone surgery for this reason and whose pathological diagnosis was PC were included in the study. As a result of the exclusion criteria, the remaining 85 patients formed the study group. Exclusion criteria were as follows: (1) patients whose pathologic diagnosis other than pancreatic adenocancer such as neuroendocrine tumor, chronic pancreatitis or intraductal papillary mucinous neoplasm; (2) patients whose psoas muscle area (PMA) and BMD measurement could not be performed on CT because of vertebral implants, motion artifacts, vertebral height loss, or scoliosis; (3) patients whose clinical follow-up and medical data were missing.

### Patients medical data

Characteristic features of patients (age, gender, height, and weight), preoperative laboratory values, presence of postoperative complications and complications type, postoperative 30- and 90-day mortality, and data on 5-year overall survival (OS) were recorded from medical data.

**Fig. 1:**
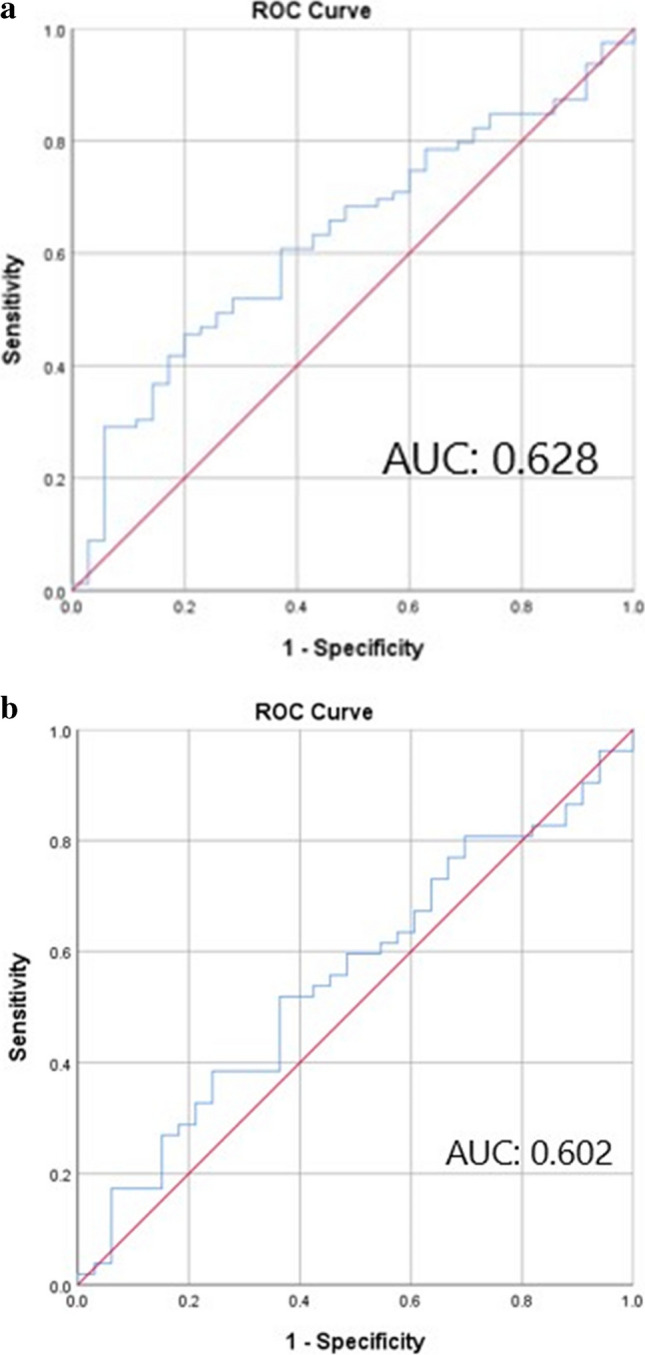
**a** ROC curv for HALP score. **b** ROC curve for PNI

Preoperative HALP score was calculated using following formula: hemoglobin (g/L) × albumin (g/L) levels × lymphocyte count (/L)/platelet count (/L). The cutoff value of the HALP score was determined according to survival status using receiver operating characteristic (ROC) analysis. The best cutoff point was 25.57 (Fig. 1a).

Preoperative PNI was calculated using the following formula: 10 × serum albumin (g/dL) + 0.005 × lymphocytes (cells/mm^3^). The best cutoff point was 38.27 (Fig. 1b).

### CT acquisition protocol

All CT imaging were performed using 64-slice (Toshiba, Aquillon 64, Japan) or 128-slice (GE, Revolution EVO, USA) multi-detector CT scanners with the following parameters: 1:0.984/1.35 pitch, automated dose modulation (200–350 mAs), 120 kVp, and 05–0.625-mm isotropic spatial resolution. Weight-adapted (1–1.5 mL/kg, maximum allowable150 mL) an iodinated intravenous contrast agent was administered with an automatic injector and all images were obtained in portal venous phases.

### Evaluation of sarcopenia on CT according to PMA

**Fig. 2:**
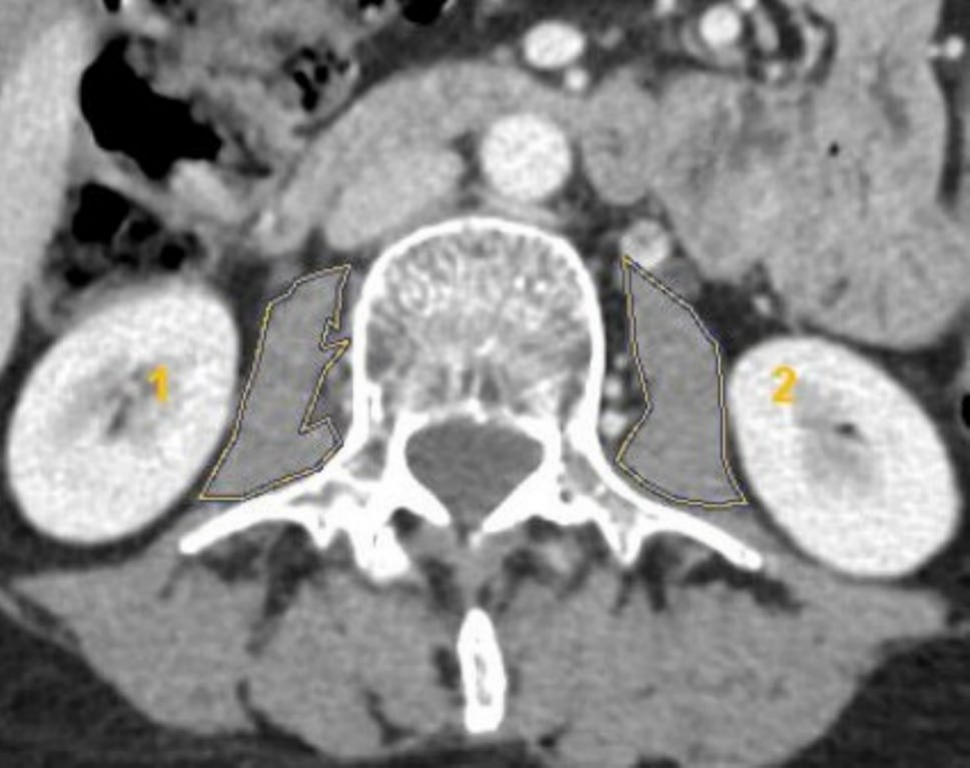
Measuring of psoas muscle areas on the axial plane CT at the level of L3 vertebra

PMA was used to evaluate the SI of the patients. PMAs were determined using the freehand method with manual delineation of muscle borders at the level of the L3 vertebra on the preoperative axial plane CT (Fig. 2). The SI was calculated by dividing the cross-sectionally obtained total PMA (sum of right and left) by the patient’s height squared. Sarcopenia was determined using the cutoff values (3.56 cm^2^/m^2^ for females, 5.40 cm^2^/m^2^ for males) mentioned in the literature [10].

### Evaluation of osteoporosis on CT according to BMD

**Fig. 3:**
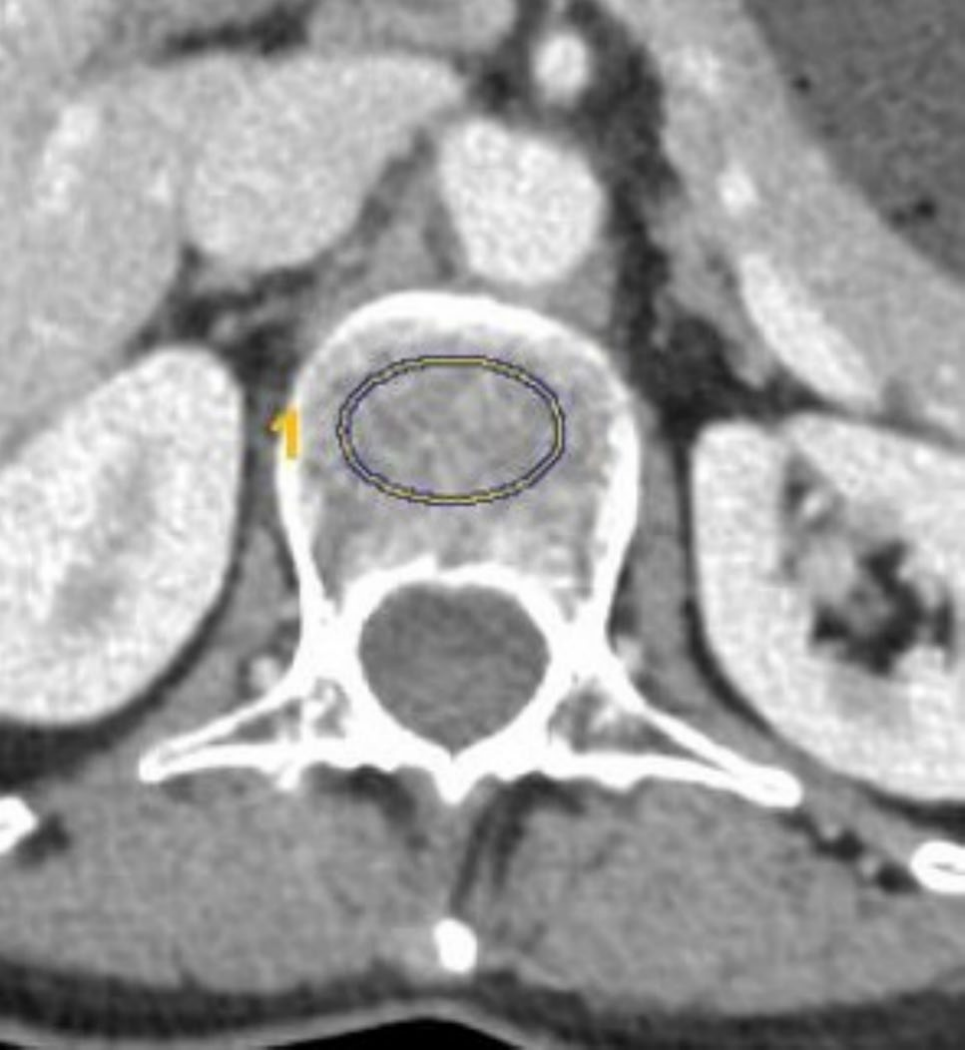
Measuring of BMD on the axial plane CT at the level of L1 vertebra

BMD was used to evaluate the osteoporosis of the patients. On the preoperative CT, BMD from axial CT were determined using the region of interest (ROI) method at the level of the L1 vertebra. Eliptic ROI were drawn in the vertebral body to measure CT attenuation values [Hounsfield Unit (HU)]. In the measurements, venous plexsus and cortical bone were avoided and sampling was made from the anterior 2/3 of the of the vertebra’s meduller component (Fig. 3). Osteoporosis was determined using the cutoff value (160 HU) mentioned in the literature [11].

### Statistical analysis

All statistical analyses were performed using SPSS statistical software, version 22.0 (Armonk, NY: IBM Corp.) and MedCalc (version 15.6.1, MedCalc Software bvba, Ostend, Belgium). For statistical significance, *p* ≤ 0.05 was accepted as the threshold value. Continuous variables were expressed as mean ± standard deviation (SD) and categorical variables were expressed as numbers and percentages (%). The chi-square test was used to evaluate the relationship between categorical variables. ROC analysis was performed to obtain the optimal cutoff values for dichotomy. Youden J index (sensitivity + specificity-1) was calculated for the optimum cutoff value for maximum sensitivity and specificity. Survival analysis was done by using the Kaplan Meier method, and differences between the curves were evaluated using the log-rank test. Survival data were used to establish a univariate Cox proportional hazards model.

## Results

The remaining 85 patients as a result of the exclusion criteria were included in the study. Clinical data of the patients are summarized in Table 1.

**Table 1:** Clinical data of the patients

Variable	All patients (*n* = 85)
Age (year)	63.74 ± 9.05
BMI (kg/cm^2^)	25.85 ± 3.72
Gender (female/male)	35 (41.17%)/50 (58.82%)
PNI	40.43 ± 5.63
Low PNI (*n* = 34)	34.95 ± 2.09
High PNI (*n* = 51)	44.08 ± 4.06
SI (cm^2^/m^2^)	3.76 ± 1.39
Sarcopenic (*n* = 53)	3.00 ± 0.76
Nonsarcopenic (*n* = 32)	5.03 ± 1.29
BMD (HU)	144.15 ± 37.93
Osteoporotic (*n* = 57)	124.14 ± 24.02
Nonosteoporotic (*n* = 28)	184.88 ± 26.73
HALP Score	35.85 ± 19.46
High HALP Score (*n* = 59)	44.21 ± 17.49
Low HALP Score (*n* = 26)	16.88 ± 4.88
Postoperative complications (yes/no)	16/69
30-day mortality (yes/no)	8/77
90-day mortality (yes/no)	11/74
5-year OS (yes/no)	50/35

^*^BMI, body mass index; *PNI*, prognostic nutritional index; *SI*, sarcopenia index; *BMD*, bone mineral density; *HU*, Hounsfield Units; *HALP*, hemoglobin, albumin, lymphocyte, platelet; *OS*, overall survival

Patients were categorized according to the presence or absence of sarcopenia and osteoporosis, high and low PNI, and HALP scores. The groups were compared in terms of the complication development rate, 30- and 90-day mortality, and OS.

**Fig. 4:**
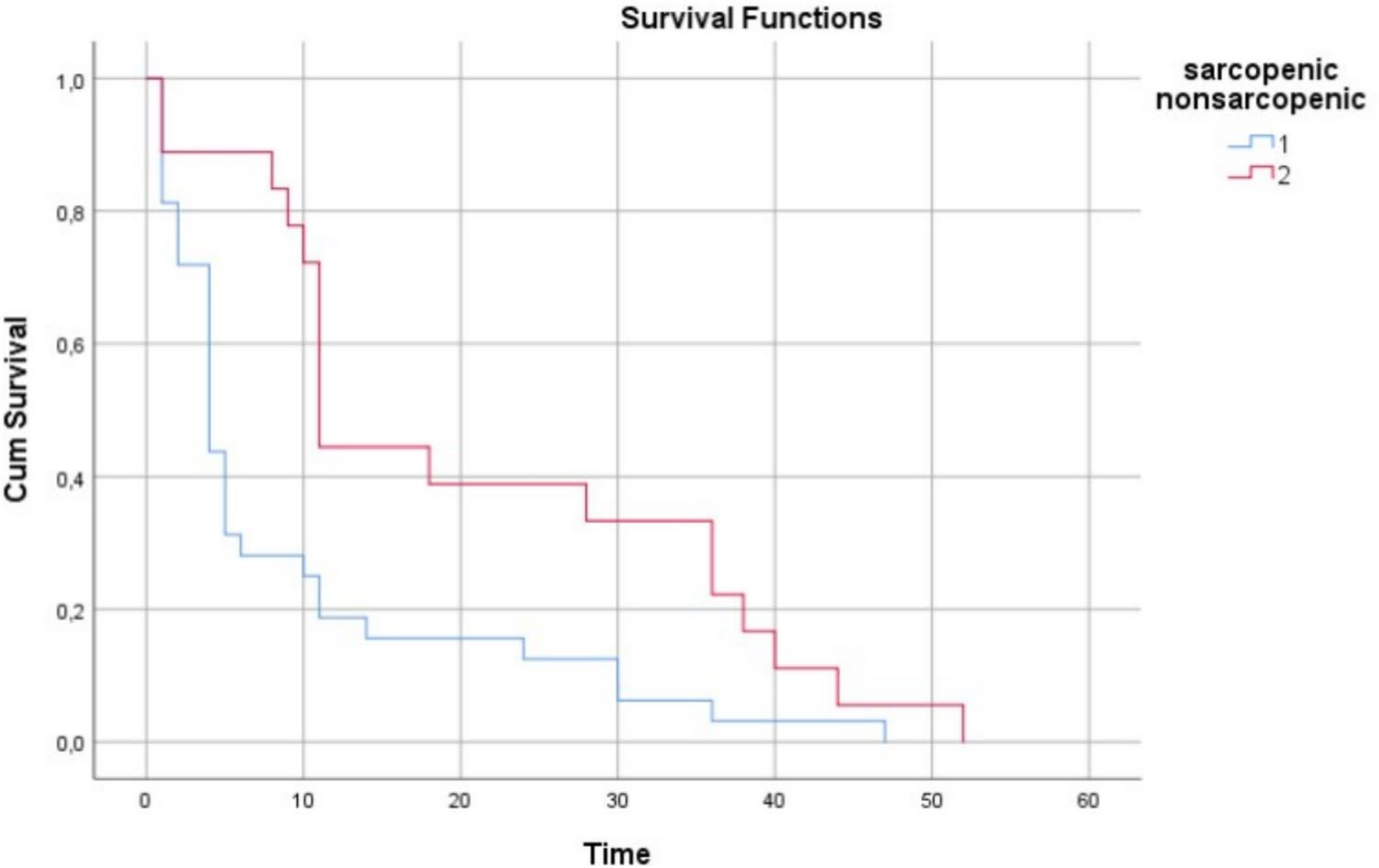
Sarcopenic and nonsarcopenic groups survival curves

Sarcopenia was detected in 53 (62.35%) of the patients included in the study, while it was not detected in 32 (37.64%) of the patients. The presence of postoperative complications, as well as 30- and 90-day mortality, did not differ between the sarcopenic and nonsarcopenic groups. Patients with sarcopenia had a 3.70 (95% CI 3.58–8.98) times higher risk of exitus than nonsarcopenic patients (*p* < 0.0001) (Fig. 4).

Osteoporosis was detected in 57 (67.05%) of the patients included in the study, while it was not detected in 28 (32.94%) of the patients. There were no differences between the groups with and without osteoporosis in terms of the presence of postoperative complications, 30- and 90-day mortality, and overall survival (OS).

Of the patients included in the study, 59 (69.41%) had a high HALP score, while 26 (30.58%) had a low HALP score. There were no differences between the low and high HALP score groups in terms of the presence of postoperative complications, 30- and 90-day mortality, and overall survival (OS).

Of the patients included in the study, 51 (60%) had a high PNI, while 34 (40%) had a low PNI. The 30-day mortality was higher in the low PNI group (*p* = 0.03). There were no differences between the low and high PNI groups in terms of the presence of postoperative complications, 90-day mortality, and overall survival (OS).

Comparisons for the groups are shown in Table 2.

**Table 2:** Comparisons of the groups

	Post-op complication Yes/no	30-day mortality Yes/no	90-day mortality Yes/no	5-year OS Yes/no
Sarcopenic (*n* = 53) Nonsarcopenic (*n* = 32)	11/42 5/27 *p* = 0.775	6/47 2/30 *p* = 0.704	9/44 2/30 *p* = 0.196	32/21 18/14 ***p* < 0.0001**
Osteoporotic (*n* = 57) Nonosteoporotic (*n* = 28)	10/47 6/22 *p* = 0.770	6/51 2/26 *p* = 0.608	9/48 2/26 *p* = 0.196	35/22 15/13 *p* = 0.09
High HALP Score (*n* = 59) Low HALP Score (*n* = 26)	9/50 7/19 *p* = 0.236	5/54 3/23 *p* = 0.696	6/53 5/21 *p* = 0.299	33/26 17/9 *p* = 0.45
High PNI (*n* = 51) Low PNI (*n* = 34)	10/41 6/28 *p* = 0.82	2/49 6/28 ***p* = 0.03**	4/47 7/27 *p* = 0.09	29/22 21/13 *p* = 0.18

^*^OS, overall survival; *HALP*, hemoglobin, albumin, lymphocyte, platelet; *PNI*, prognostic nutritional index

## Discussion

In this study, we evaluated the prognostic impact of preoperative PNI, HALP score, SI, and BMD values on postoperative complications and survival in patients with resectable PC, while no differences were found between the sarcopenic and non-sarcopenic groups in terms of the presence of postoperative complications, 30-day, and 90-day mortality. Patients with sarcopenia had a 3.70 times higher risk of death within 5 years compared to non-sarcopenic patients. No differences were found between the osteoporotic and non-osteoporotic groups, as well as between the high and low HALP score groups, in terms of the presence of postoperative complications, 30-day and 90-day mortality, and 5-year overall survival. The 30-day mortality was higher in the low PNI group, whereas there were no differences between the low and high PNI groups in terms of the presence of postoperative complications, 90-day mortality, and 5-year overall survival.

PC patients are at risk of sarcopenia due to both malignancy-related factors and nutritional deterioration caused by pain. Sarcopenia has been identified as a potential risk factor for morbidity and mortality in patients with gastrointestinal malignancies [12]. A meta-analysis evaluating 23 studies and 5888 patients investigating the association between preoperative sarcopenia and the prognosis of PC after curative-intent surgery found that preoperative sarcopenia was significantly associated with worse OS [13]. However, the study also found that the development of major postoperative complications was not associated with sarcopenia. In our study, similar to the literature, we did not find an association between the presence of postoperative complications and sarcopenia, but 5-year OS was lower in the sarcopenic group. The mechanism behind the association between sarcopenia and poor prognosis is not well understood. However, sarcopenia is a catabolic process that is accompanied by impaired immune response [14]. It is suggested that both systemic immune response and nutritional decline may affect treatment tolerance and response [14].

In addition to sarcopenia, low BMD is also an important factor in cancer patients. It is believed that low BMD is associated with a higher risk of falls, fractures, institutionalization, death, and prognosis [15]. Watanabe et al., in their meta-analysis evaluating the prognostic significance of preoperative low bone mineral density in digestive cancer patients, found that low BMD was associated with poor prognosis in colorectal, esophageal, hepatic, and bile duct cancers, but it was ineffective in PC [15]. In our study, we did not find an association between osteoporosis and the presence of postoperative complications, mortality, or 5-year OS. The impact of osteoporosis on poor prognosis in digestive cancers remains unclear. It is suggested that osteoporosis is associated with sarcopenia and therefore considered a poor prognostic factor. However, our study’s findings do not support this. While sarcopenia was found to be a prognostic factor, osteoporosis was not. Further studies are warranted to investigate in detail the relationship between osteoporosis and sarcopenia and their prognostic values in patients with digestive tract cancers.

HALP score is considered a novel marker reflecting both systemic inflammation and nutritional status [16]. In one study, a low HALP score was reported as a risk factor for shorter OS in resected PC patients [16]. Another study found that a low HALP score was a risk factor for postoperative complications and lower OS [4]. However, contrary to these findings, there is also a study indicating that low HALP score did not pose a risk for complications other than pancreatic fistula and OS [17]. We think that the impact of HALP score on postoperative complications and OS is not yet clear and needs to be supported by further studies.

PNI is a score that reflects the immunonutritional status. Poor nutritional status is associated with increased infection risk and delayed wound healing, while systemic inflammation can affect clinical outcomes through tumor proliferation, survival, angiogenesis, and metastasis. Therefore, studies have been conducted on the prognostic importance of PNI in both PC and other malignancies. In patients with resectable PC, a high PNI score has been shown to be associated with longer OS [18, 19]. It has also been linked to postoperative complications, particularly pancreatic fistula [20]. In our study, we did not find an association between low PNI and the presence of postoperative complications or 5-year OS. However, it was associated with early-term (30 days) mortality. One of the components in the PNI formula is albumin. As the half-life of albumin is approximately 20 days, it reflects mid- and long-term nutritional status [20]. High 30-day mortality in low PNI appears to be associated with low albumin levels. In patients with low preoperative albumin levels, albumin supplementation may reduce early mortality. Considering the half-life of albumin, its monitoring and supplementation when necessary should be kept in mind during the early postoperative period.

Our study had some limitations. The first limitation is that it is a retrospective and single-center study. A second limitation is the relatively small number of patients. However, a strength of our study is the evaluation of both early and long-term outcomes with different nutritional indicators in the same patient group. While the most important factors influencing morbidity, mortality, and patient prognosis in PC are the tumor stage and resectability, these are not the only considerations. Demographic characteristics, comorbid conditions, and the status of neoadjuvant and adjuvant treatments also play a role in prognosis. The inability to evaluate these factors is a limitation of the study.

In conclusion, PNI may be used as prognostic data for early postoperative mortality, while sarcopenia may be indicative of 5-year OS in patients with resectable PC. Our results suggest that providing nutritional support may potentially improve prognosis. Future studies, in which other factors effective in prognosis are evaluated together with nutritional status, will show more information on this subject.

## Data Availability

No datasets were generated or analysed during the current study.
